# Supplementary material for: Design, Synthesis, and Anti-Proliferative Action of Purine/Pteridine-Based Derivatives as Dual Inhibitors of EGFR and BRAFV600E
Source: Pharmaceuticals (Basel). 2023 May 8;16(5):716. doi: 10.3390/ph16050716 (PMC10223936; doi:10.3390/ph16050716)
Supplement: Supplementary file 1 [file pharmaceuticals-16-00716-s001.zip › pharmaceuticals-2347144-supplementary.pdf]

# Design, Synthesis, and antiproliferative action of purine/pteridine-based derivatives as dual inhibitors of EGFR and BRAF$^{V600E}$

Samar A. El-Kalyoubi[1], Hesham A. M. Gomaa[2], Elshimaa M. N. Abdelhafez[3], Mohamed Ramadan[4], Fatimah Agili[5], Bahaa G. M. Youssif[6]

[1]Department of Pharmaceutical Organic Chemistry, Faculty of Pharmacy, Port Said University, 42511 Port Said, Egypt; [2]Department of Pharmacology, College of Pharmacy, Jouf University, Sakaka, 72341, Aljouf, Saudi Arabia; [3]Medicinal chemistry department, Faculty of Pharmacy, Minia University Minia, Egypt, 61519; [4]Pharmaceutical Organic Chemistry Department, Faculty of Pharmacy, Al-Azhar University, Assiut Branch, Assiut, Egypt; [5]Chemistry Department, Faculty of Science (Female Section), Jazan University, Jazan 82621, Saudi Arabia; [5]Pharmaceutical Organic Chemistry Department, Faculty of Pharmacy, Assiut University, Assiut 71526, Egypt.

**To whom correspondence should be addressed:*

**Samar A. El-Kalyoubi,** Ph.D. Department of Pharmaceutical Organic Chemistry, Faculty of Pharmacy, Port Said University, 42511 Port Said, Egypt.

**E-mail address**: S.elkalyoubi@hotmail.com

**Bahaa G. M. Youssif**, Ph.D. Pharmaceutical Organic Chemistry Department, Faculty of Pharmacy, Assiut University, Assiut 71526, Egypt.

**Tel**.: (002)-01098294419

**E-mail address**: bahaa.youssif@pharm.aun.edu.eg, bgyoussif2@gmail.com

## Table of Contents


| | Figure number | Page number |
|---|---|---|
| $^{1}H$ NMR, $^{13}C$ NMR and IR of **2,7-dibromo-3'-methyl-7',9'-dihydrospiro[fluorene-9,8'-purine]-2',6'(1'*H*,3'*H*)-dione (5a)** | **Figure S1** | 3,4 |
| $^{1}H$ NMR, $^{13}C$ NMR and IR of **2,7-dibromo-3'-ethyl-7',9'-dihydrospiro[fluorene-9,8'-purine]-2',6'(1'*H*,3'*H*)-dione (5b)** | **Figure S2** | 4,5 |
| $^{1}H$ NMR, $^{13}C$ NMR and IR of **2,7-dibromo-3'-methyl-2'-thioxo-2',3',7',9'-tetrahydrospiro[fluorene-9,8'-purin]-6'(1'*H*)-one (5c)** | **Figure S3** | 6,7 |
| $^{1}H$ NMR, $^{13}C$ NMR and IR of **3'-benzyl-2,7-dibromo-7',9'-dihydrospiro[fluorene-9,8'-purine]-2',6'(1'*H*,3'*H*)-dione (5d)** | **Figure S4** | 7,8 |
| $^{1}H$ NMR and $^{13}C$ NMR of **2,7-dibromo-3'-(2-chlorobenzyl)-7',9'-dihydrospiro[fluorene-9,8'-purine]-2',6'(1'*H*,3'*H*)-dione (5e)** | **Figure S5** | 9,10 |
| $^{1}H$ NMR, $^{13}C$ NMR and IR of **8-methylacenaphtho[1,2-*g*]pteridine-9,11(8*H*,10*H*)-dione (7a)** | **Figure S6** | 10,11 |
| $^{1}H$ NMR,$^{13}C$ NMR and IR of **8-ethylacenaphtho[1,2-g]pteridine-9,11(8H,10H)-dione (7b)** | **Figure S7** | 12,13 |
| $^{1}H$ NMR and IR of **8-methyl-9-thioxo-9,10-dihydroacenaphtho[1,2-*g*]pteridin-11(8*H*)-one (7c)** | **Figure S8** | 13 |
| $^{1}H$ NMR, $^{13}C$ NMR and IR of **8-benzylacenaphtho[1,2-*g*]pteridine-9,11(8*H*,10*H*)-dione (7d)** | **Figure S9** | 14,15 |
| $^{1}H$ NMR, $^{13}C$ NMR and IR of **8-(2-chlorobenzyl)acenaphtho[1,2-*g*]pteridine-9,11(8*H*,10*H*)-dione (7e)** | **Figure S10** | 15,16 |
| $^{1}H$ NMR, $^{13}C$ NMR and IR of **acenaphtho[1,2-*g*]pteridine-9,11(8*H*,10*H*)-dione (7f)** | **Figure S11** | 17,18 |
| Docking Study | **Figure S12** | 18,19 |
| Docking Study | **Figure S13** | 19,20 |

**Figure S1:** $^{1}$H NMR, $^{13}$C NMR and IR of **2,7-dibromo-3'-methyl-7',9'-dihydrospiro[fluorene-9,8'-purine]-2',6'(1'*H*,3'*H*)-dione (5a)**

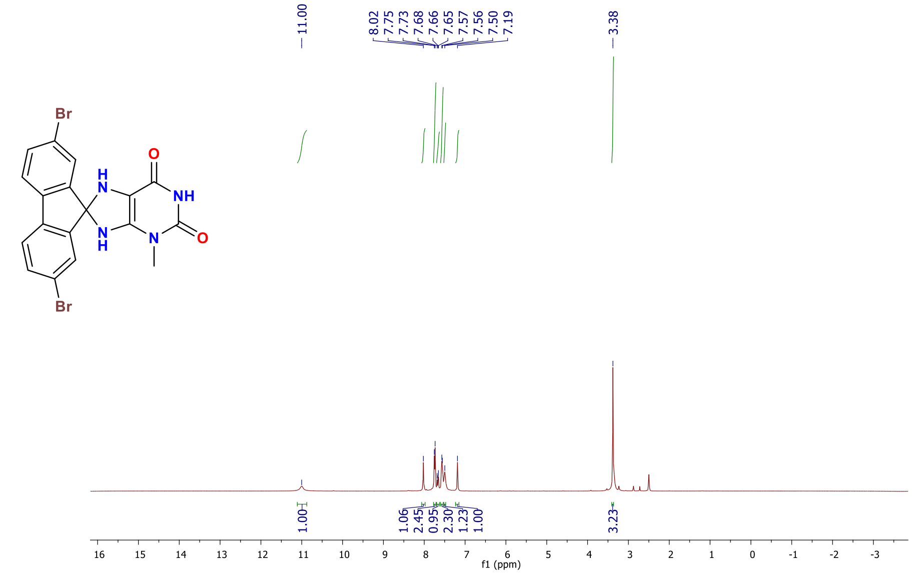


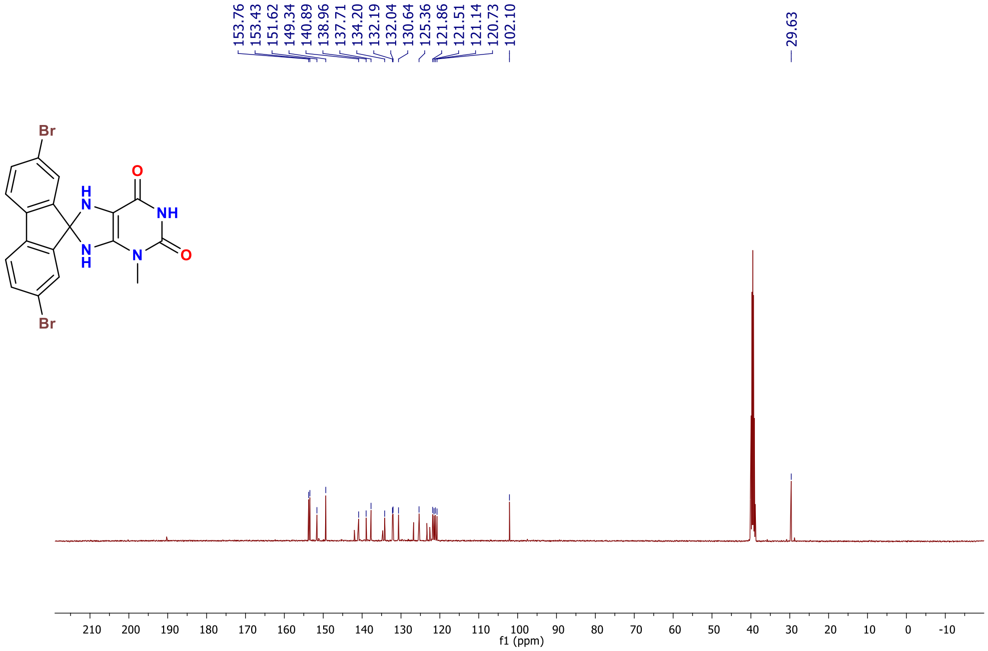

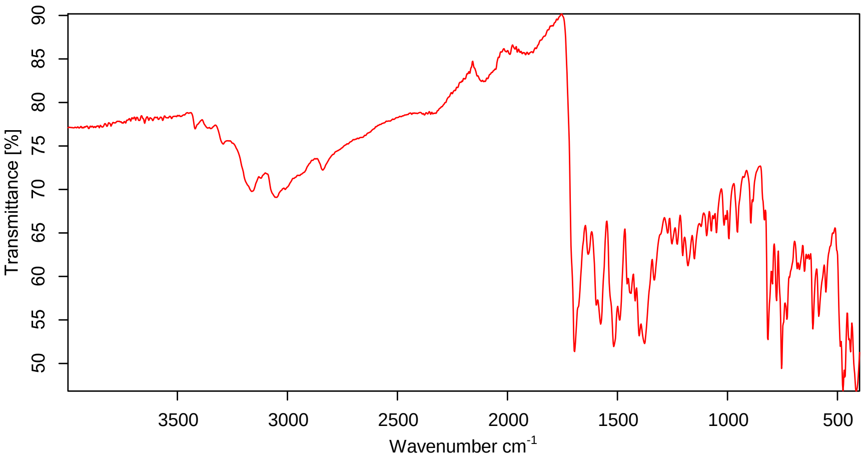


**Figure S2:**$^{1}$H NMR, $^{13}$C NMR and IR of **2,7-dibromo-3'-ethyl-7',9'-dihydrospiro[fluorene-9,8'-purine]-2',6'(1'*H*,3'*H*)-dione (5b)**

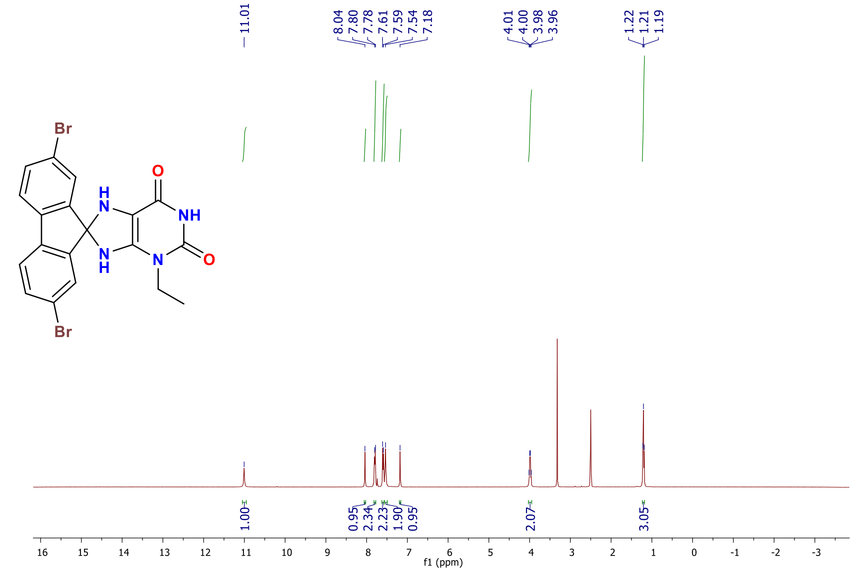

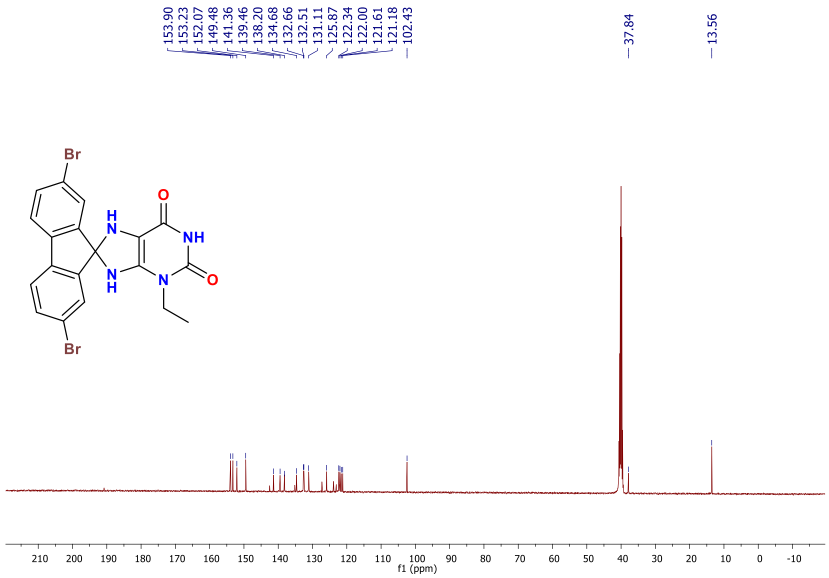
153.90
153.23
152.07
149.48
141.36
139.46
138.20
134.68
132.66
132.51
131.11
125.87
122.34
122.00
121.61
121.18
102.43
37.84
13.56
Br
H
N
O
NH
N
H
N
O
Br
210 200 190 180 170 160 150 140 130 120 110 100 90 80 70 60 50 40 30 20 10 0 -10
f1 (ppm)

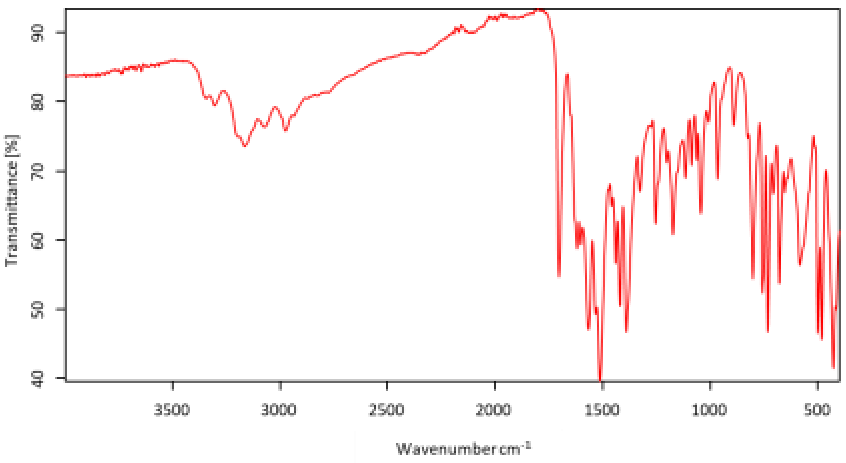
Transmittance [%]
90
80
70
60
50
40
3500 3000 2500 2000 1500 1000 500
Wavenumber cm-1

**Figure S3:**$^{1}$H NMR, $^{13}$C NMR and IR of **2,7-dibromo-3'-methyl-2'-thioxo-2',3',7',9'-tetrahydrospiro[fluorene-9,8'-purin]-6'(1'*H*)-one (5c)**

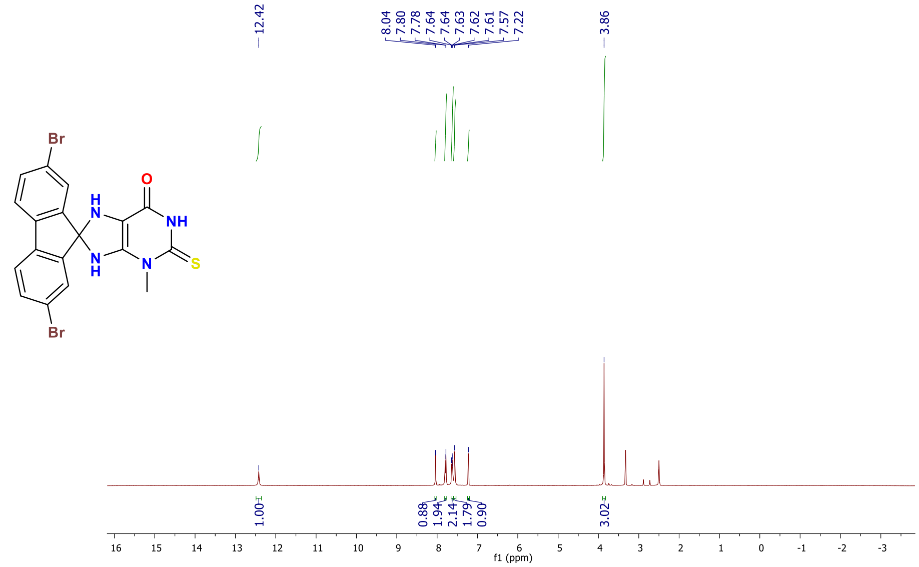


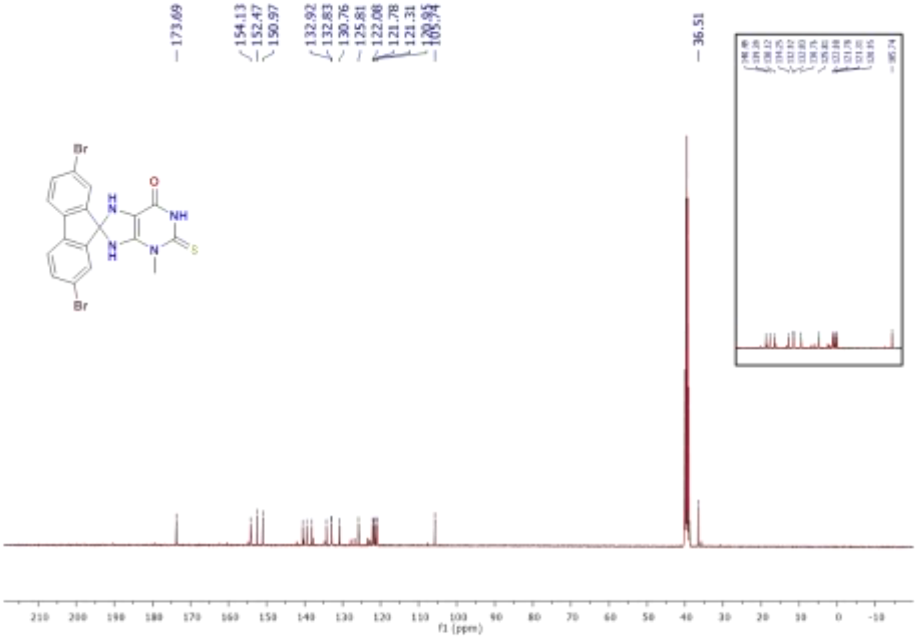

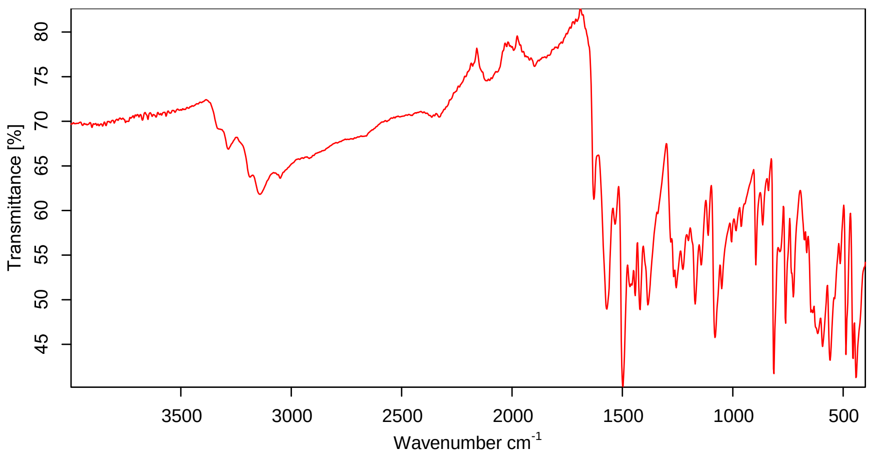


**Figure S4:**$^{1}$H NMR, $^{13}$C NMR and IR of **3'-benzyl-2,7-dibromo-7',9'-dihydrospiro[fluorene-9,8'-purine]-2',6'(1'*H*,3'*H*)-dione (5d)**

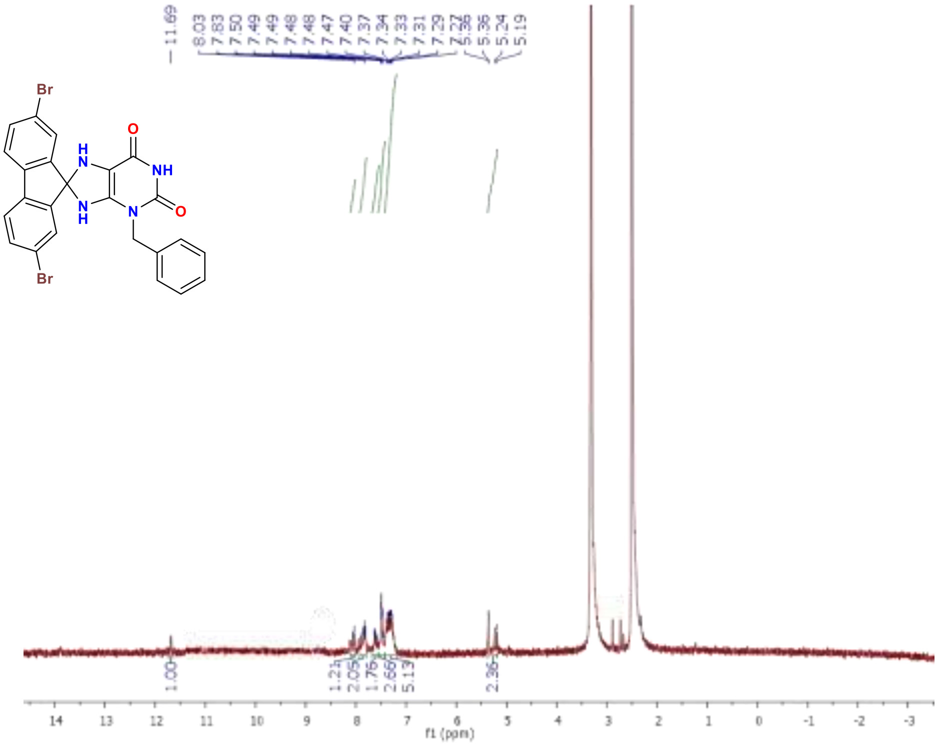

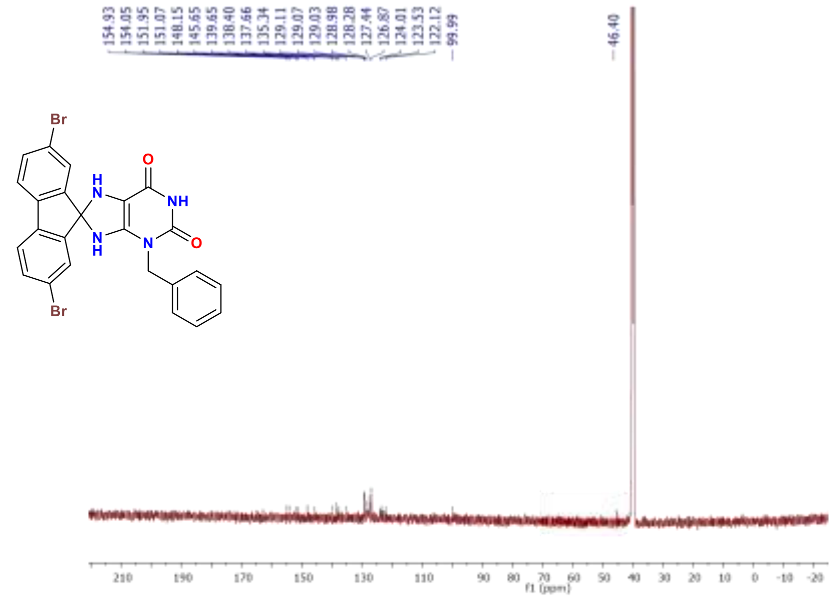

154.93
154.05
151.95
151.07
148.15
145.65
139.65
138.40
137.66
135.34
129.11
129.07
129.03
128.98
128.28
127.44
126.87
124.01
123.53
122.12
99.99
46.40
Br
Br
O
O
NH
NH
NH
N
f1 (ppm)


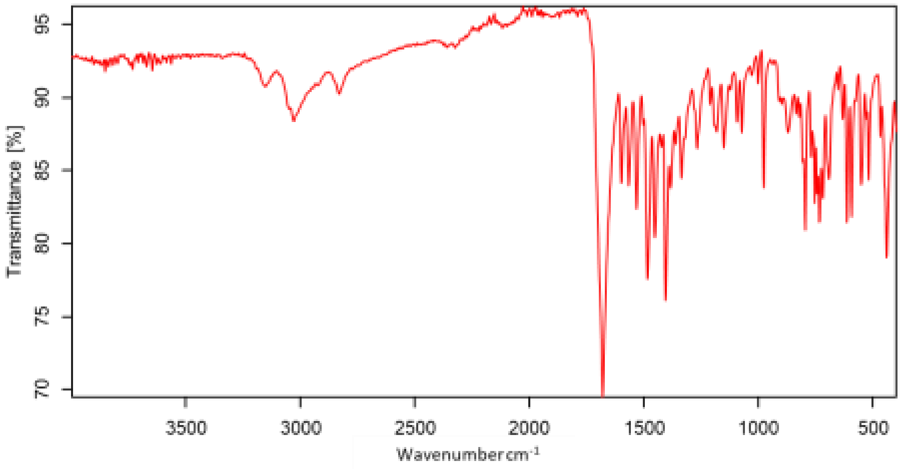

Transmittance [%]
Wavenumber cm-1

**Figure S5:** $^{1}$H NMR, $^{13}$C NMR and IR of **2,7-dibromo-3'-(2-chlorobenzyl)-7',9'-dihydrospiro[fluorene-9,8'-purine]-2',6'(1'*H*,3'*H*)-dione (5e)**

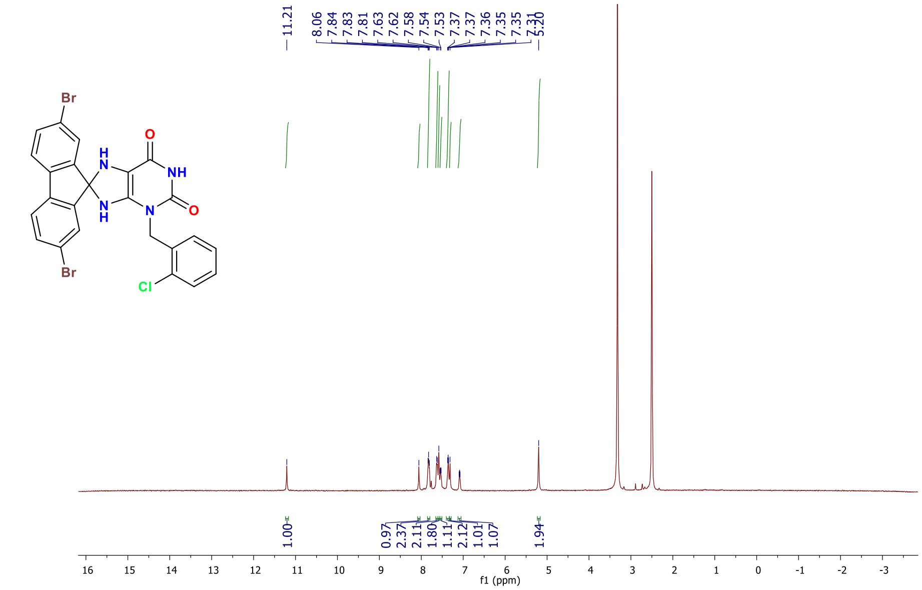


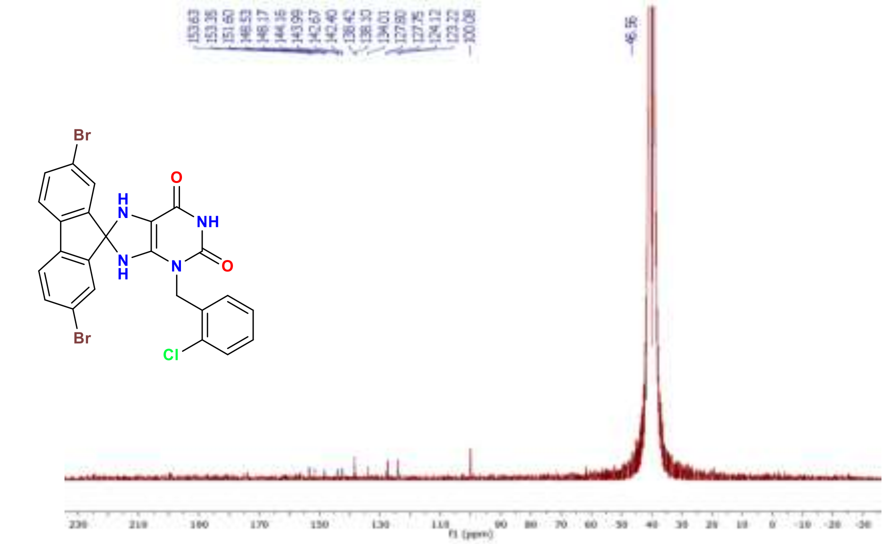

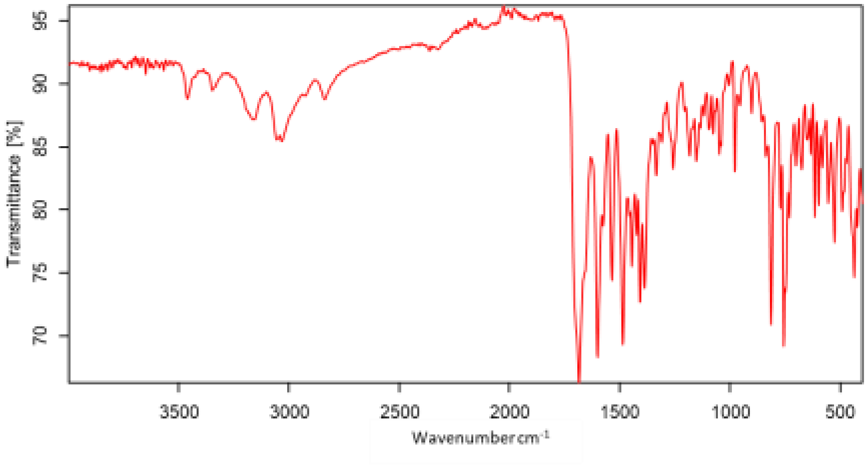


**Figure S6:** $^{1}H$ NMR, $^{13}C$ NMR and IR of **8-methylacenaphtho[1,2-*g*]pteridine-9,11(8*H*,10*H*)-dione (7a)**

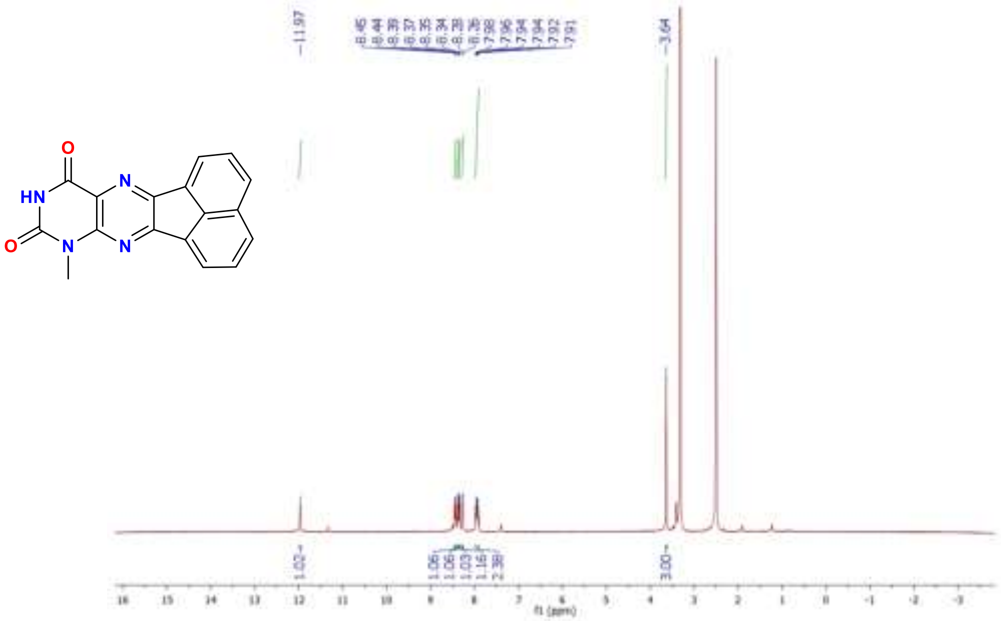

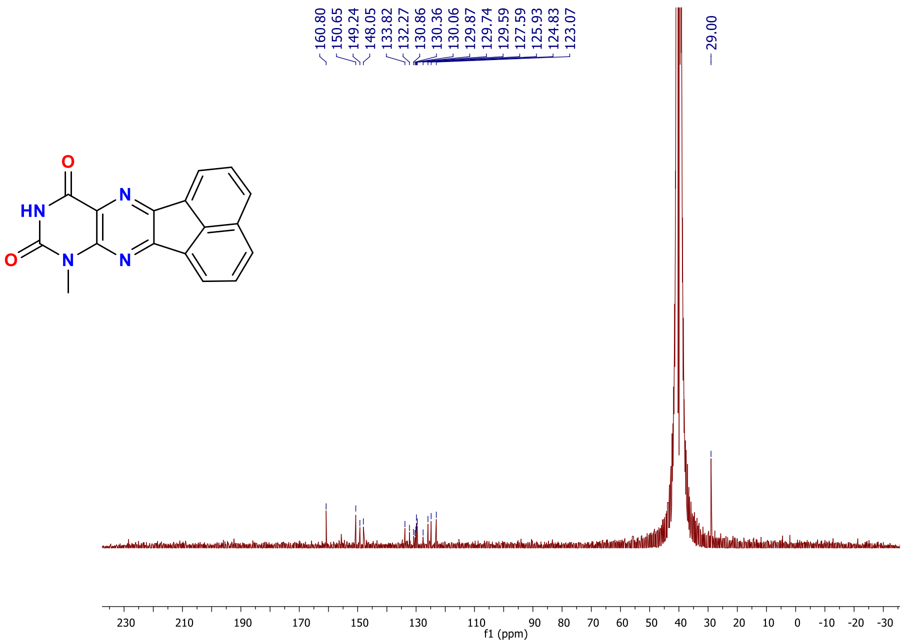
160.80
150.65
149.24
148.05
133.82
132.27
130.86
130.36
130.06
129.87
129.74
129.59
127.59
125.93
124.83
123.07
29.00
230 210 190 170 150 130 110 90 80 70 60 50 40 30 20 10 0 -10 -20 -30
f1 (ppm)

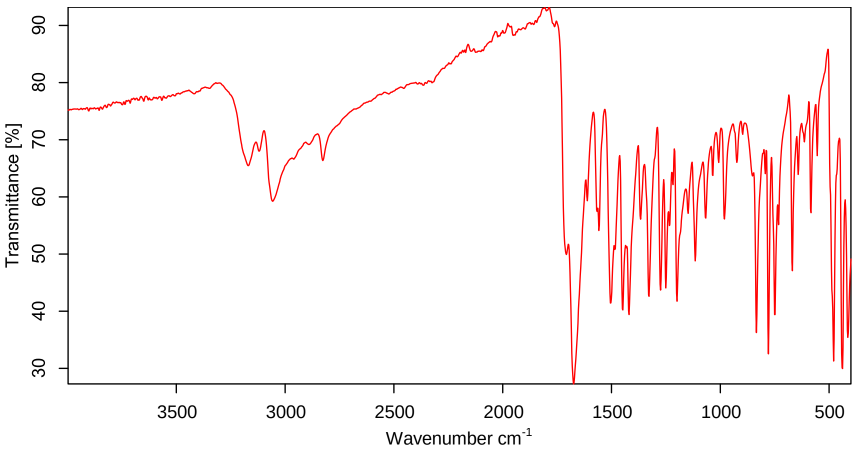
Transmittance [%]
90 80 70 60 50 40 30
3500 3000 2500 2000 1500 1000 500
Wavenumber cm-1

**Figure S7:**$^{1}$H NMR, $^{13}$C NMR and IR of **8-ethylacenaphtho[1,2-g]pteridine-9,11(8*H*,10*H*)-dione (7b)**

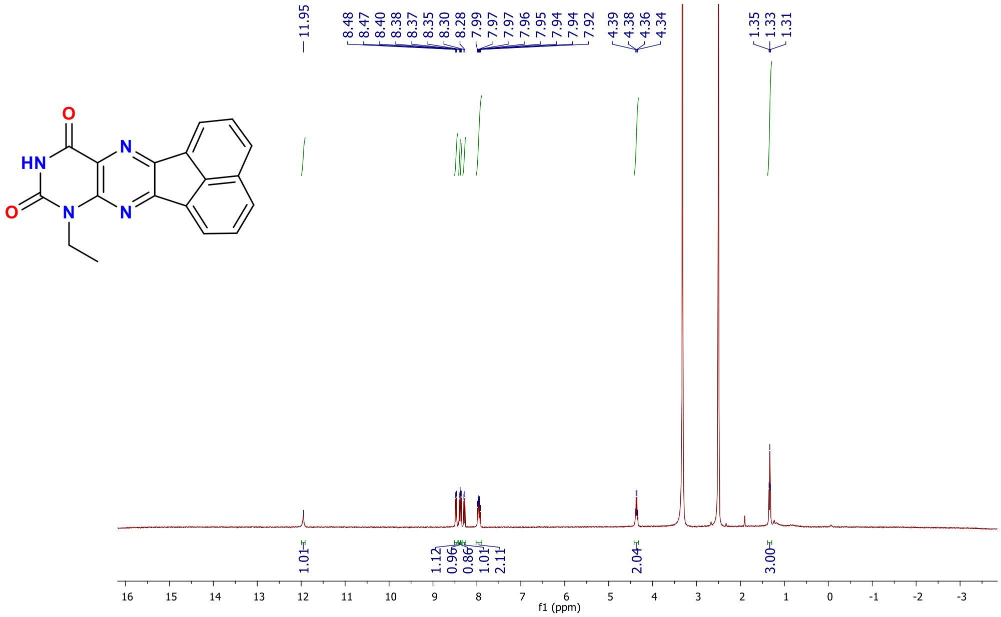

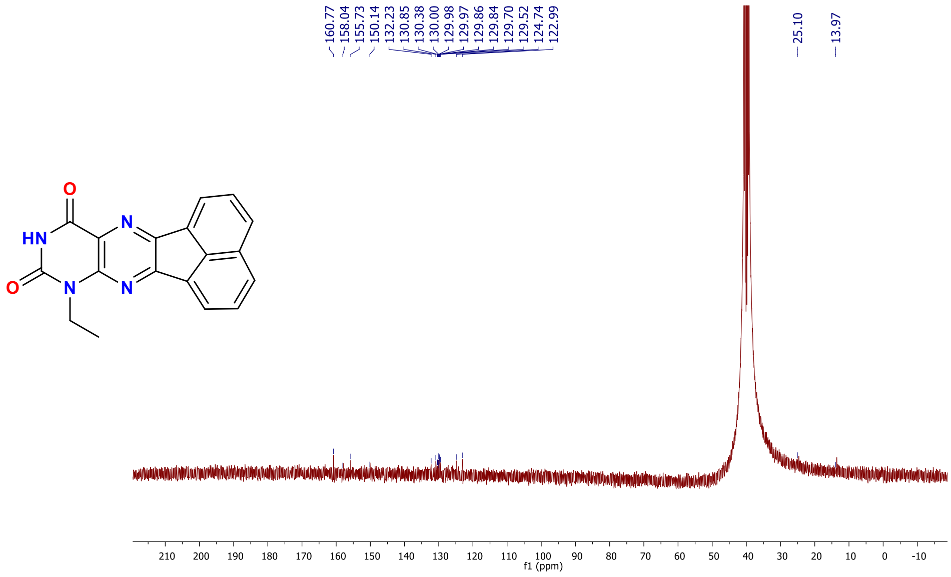

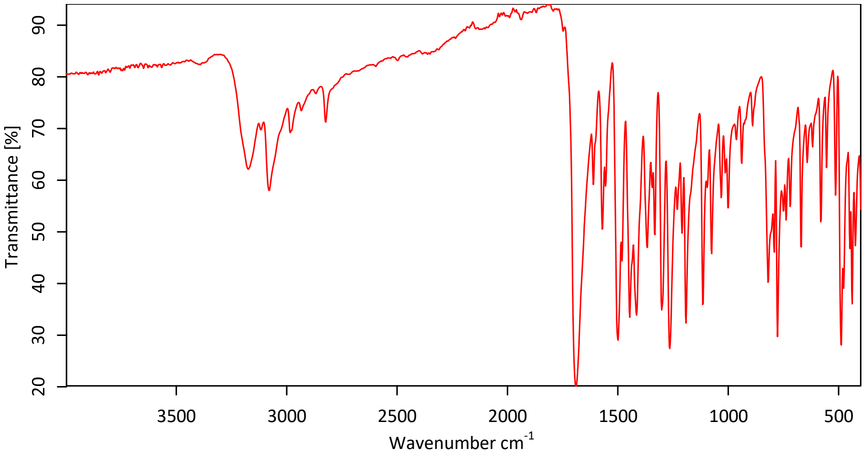


**Figure S8:**$^{1}$H NMR and IR of **8-methyl-9-thioxo-9,10-dihydroacenaphtho[1,2-*g*]pteridin-11(8*H*)-one (7c)**

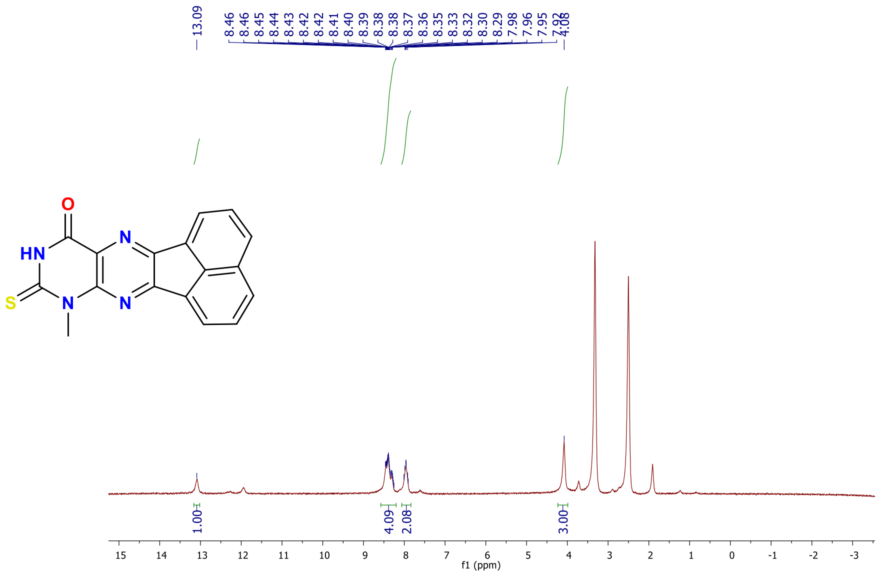


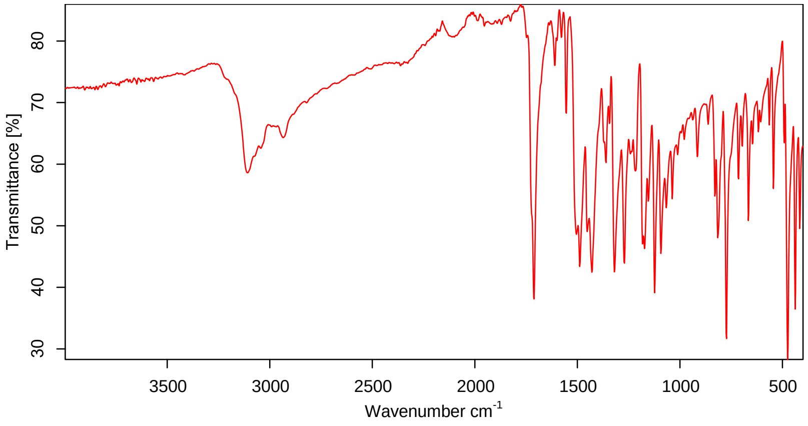

**Figure S9:**$^{1}$H NMR, $^{13}$C NMR and IR spectra of **8-benzylacenaphtho[1,2-*g*]pteridine-9,11(8*H*,10*H*)-dione (7d)**

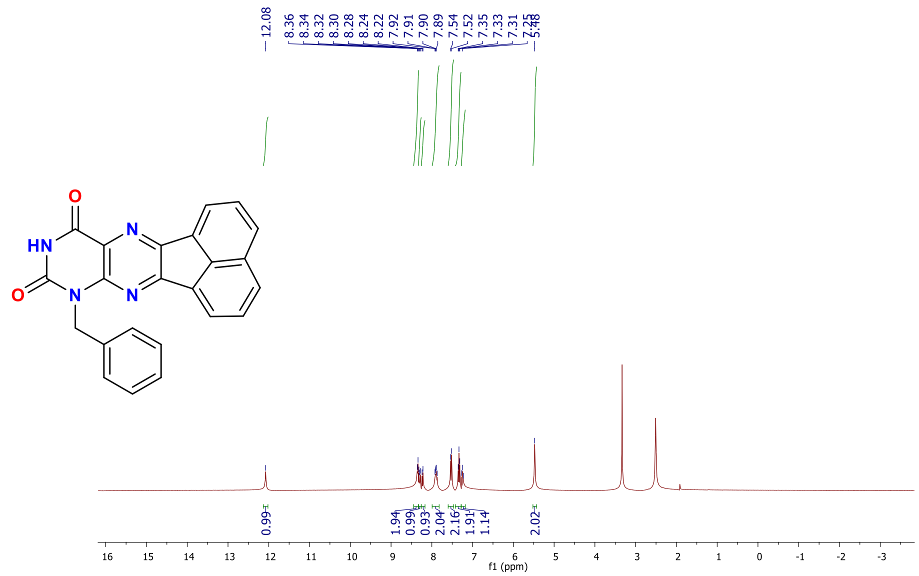


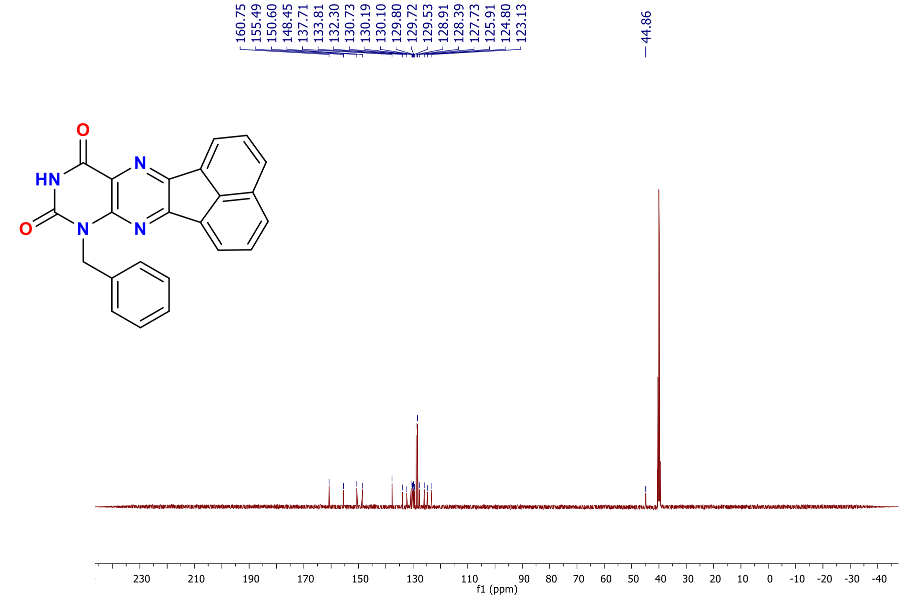

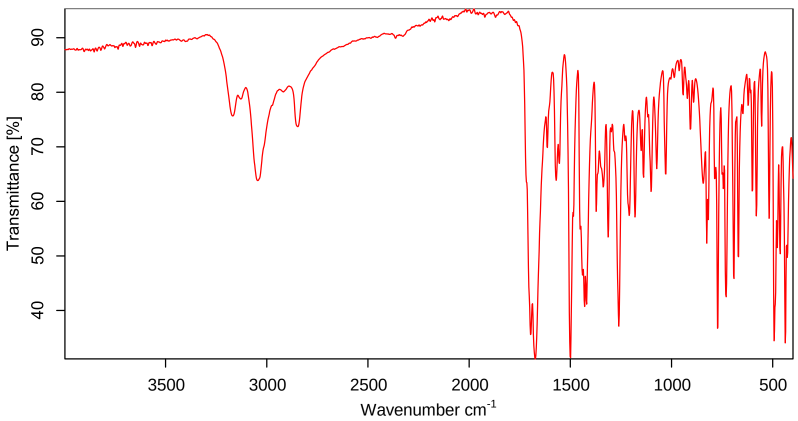


**Figure S10:**$^{1}$H NMR, $^{13}$C NMR and IR of **8-(2-chlorobenzyl)acenaphtho[1,2-*g*]pteridine-9,11(8*H*,10*H*)-dione (7e)**

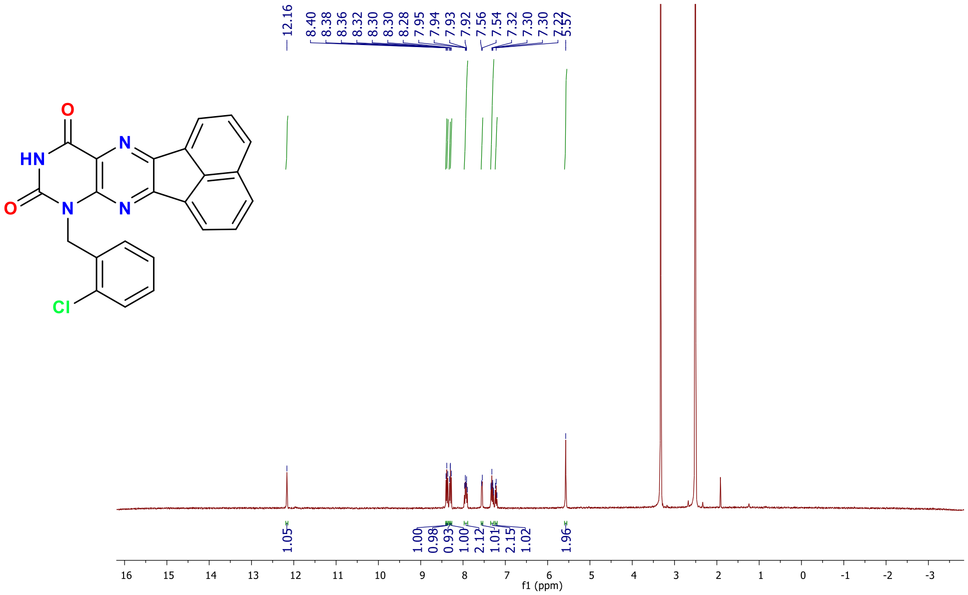

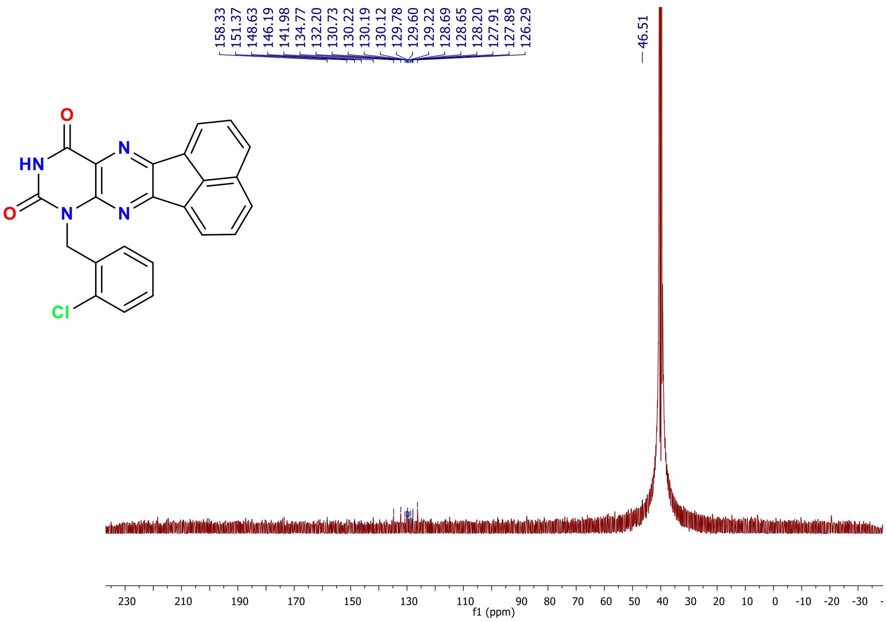

158.33
151.37
148.63
146.19
141.98
134.77
132.20
130.73
130.22
130.19
130.12
129.78
129.60
129.22
128.69
128.65
128.20
127.91
127.89
126.29
46.51
O
HN
O
N
N
N
Cl
230 210 190 170 150 130 110 90 80 70 60 50 40 30 20 10 0 -10 -20 -30
f1 (ppm)


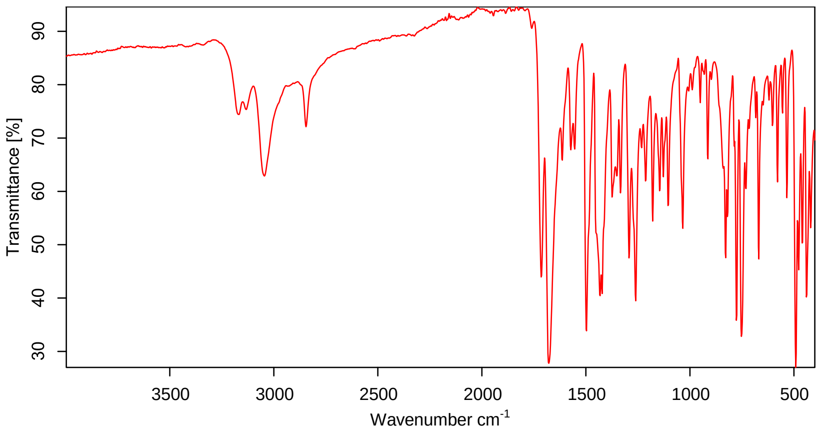

Transmittance [%]
90 80 70 60 50 40 30
3500 3000 2500 2000 1500 1000 500
Wavenumber cm-1

**Figure S11:**$^{1}$H NMR, $^{13}$C NMR and IR of **acenaphtho[1,2-*g*]pteridine-9,11(8*H*,10*H*)-dione (7f)**

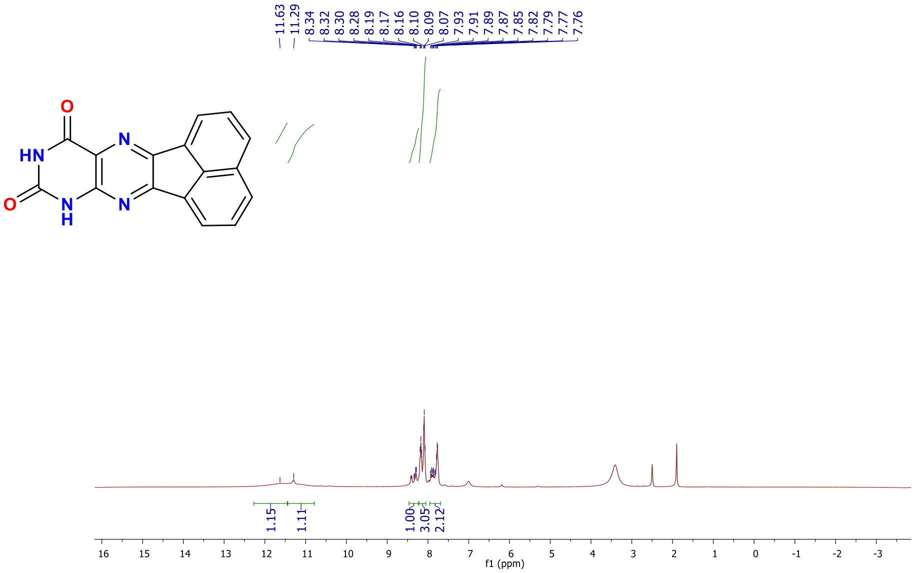


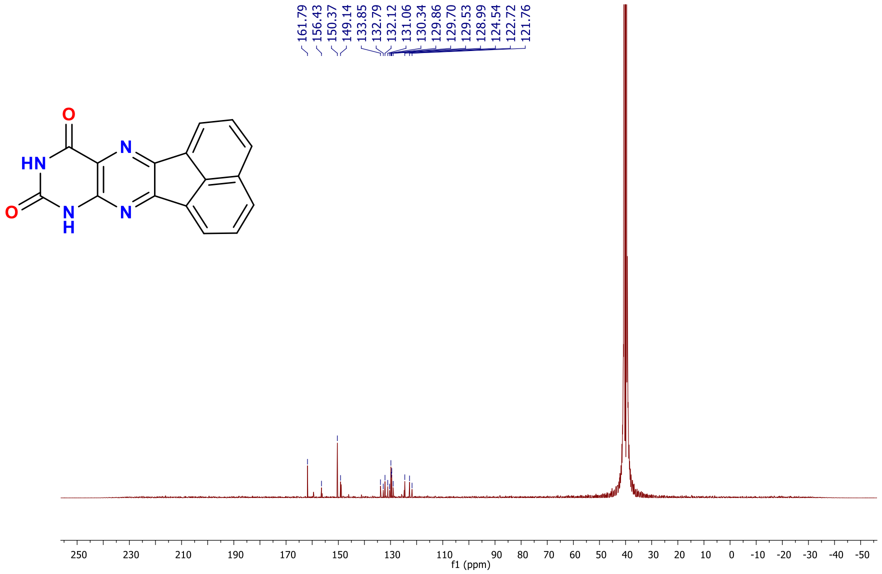

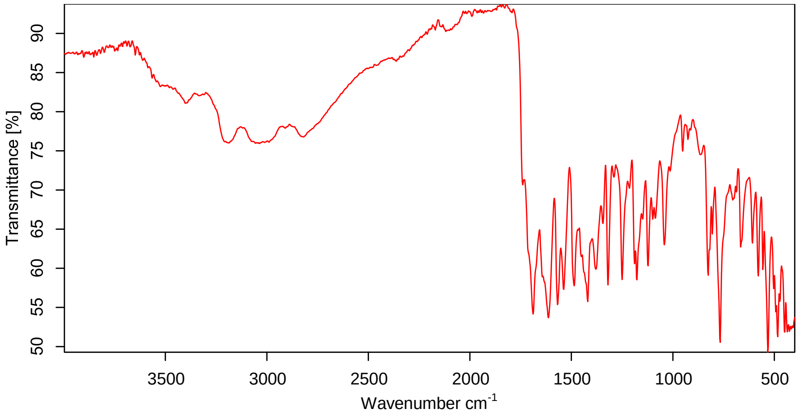


## 2. Docking Study

5c

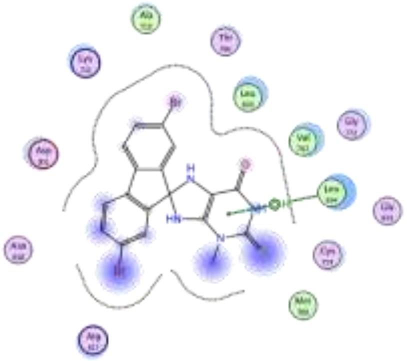

(5d)

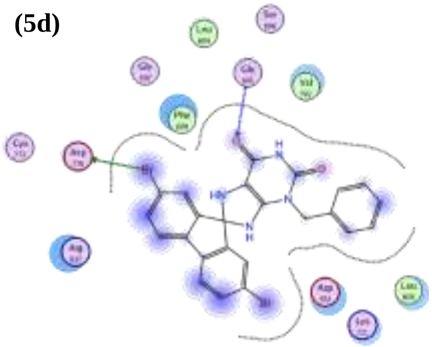

(5e)

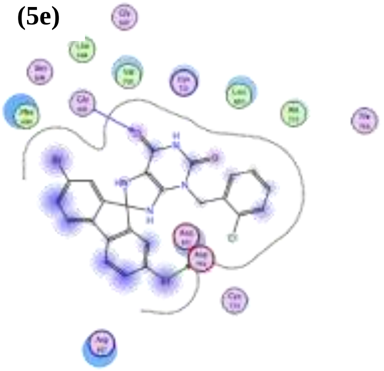

5c

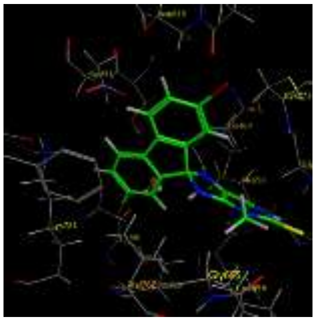

(5d)

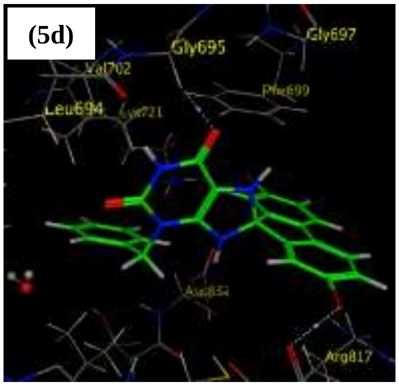


(5e)

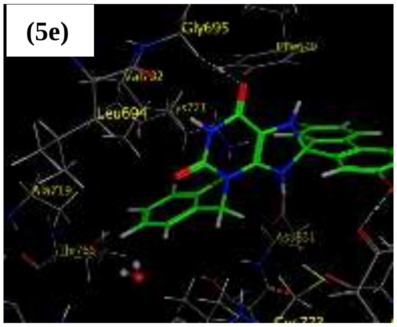

(7a)

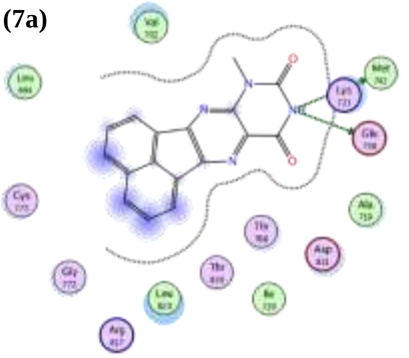

7c

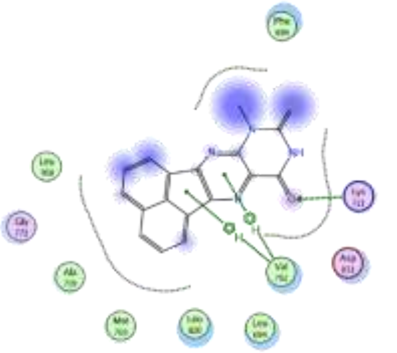

(7a)

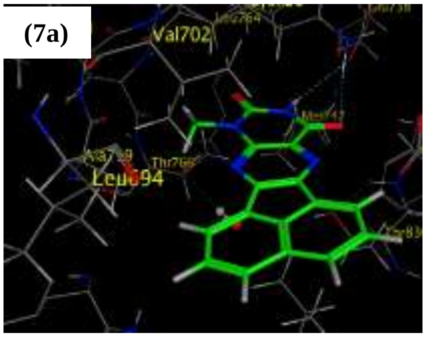


7c

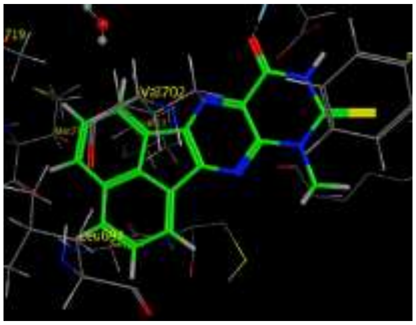


**Fig. S12.** 2D and 3D Interaction diagram of **5c,d,e & 7a,c** within EGFR (PDB ID: 1M17

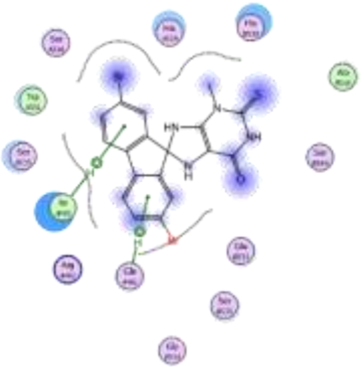

(5d)

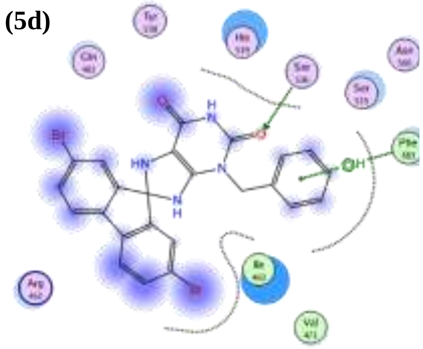

(5e)

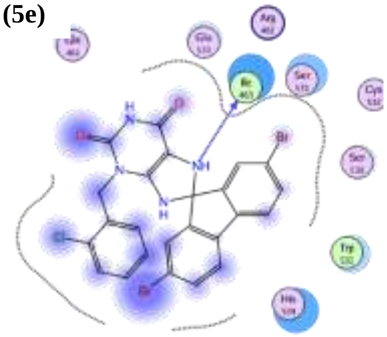

5c

5c

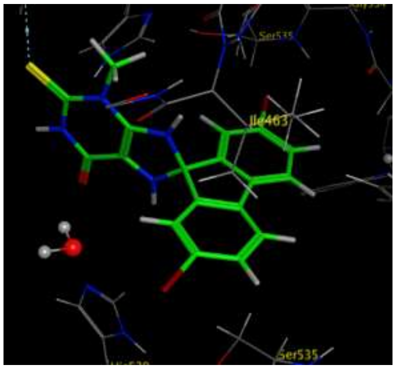


(5d)

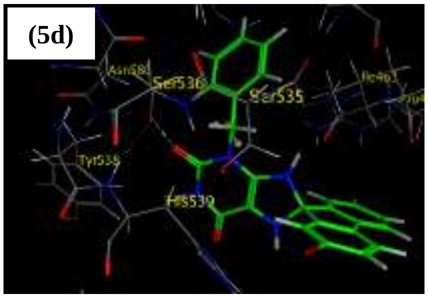


(5e)

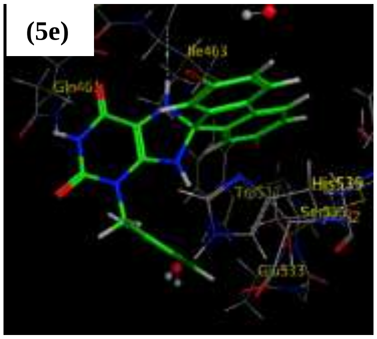

(7a)

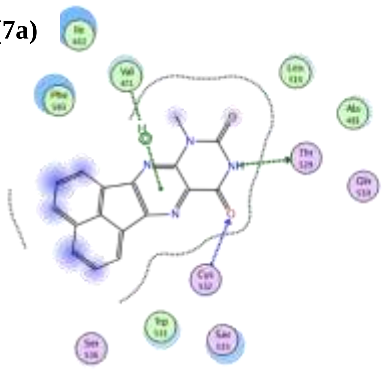

7c

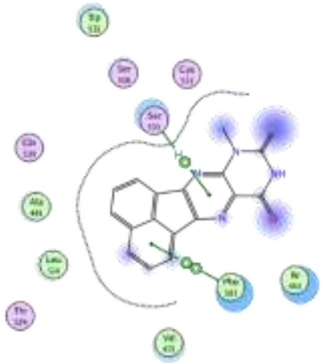

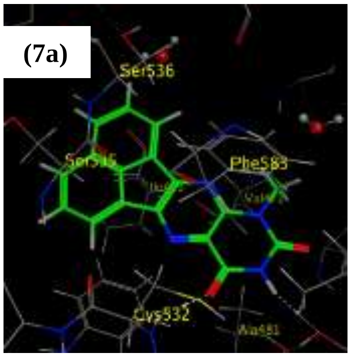


7c

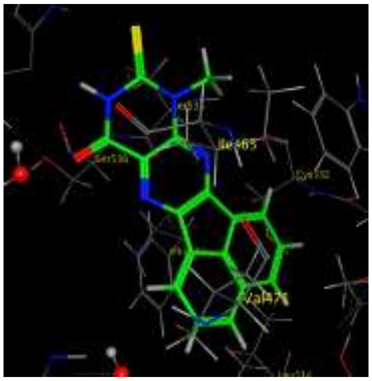


**Fig.S13.** 2D and 3D Interaction diagram of **5d,e & 7a** within BRAF$^{V600E}$ (PDB ID: 5JRQ)

## Suppl. File S1

## 4. EXPERIMENTAL

### 4.1. Chemistry

**General details**

All melting points were determined by an Electrothermal Mel.-Temp. II apparatus and were uncorrected. Element analyses were performed at Regional Center for Mycology and Biotechnology at Al-Azhar University. Mass spectra were recorded on DI-50 unit of Shimadzu GC/MS-QP 5050A at the Regional Center for Mycology and Biotechnology at Al-Azhar University. The proton nuclear magnetic resonance ($^{1}$H-NMR) spectra were recorded on Bruker 400 MHz Spectrometer and $^{13}$C-NMR spectra were run at 125 MHz in dimethylsulfoxide (DMSO-$d_6$) and TMS as an internal standard, Applied Nucleic Acid Research Center, Zagazig University, Egypt. All new compounds gave corresponding elemental analyses (C, H, N, typically ±0.3%). All reactions were monitored by TLC using precoated plastic sheets silica gel (Merck 60 F254) and spots were visualized by irradiation with UV light (254 nm). The used solvent system was chloroform: methanol (9:1) and ethyl acetate: toluene (1:1).

## 4.2. Biological evaluation

### 4.2.1. Cytotoxic activity using MTT Assay and evaluation of $IC_{50}$

#### 4.2.1.1. MTT assay

MTT assay was carried out to study the effect of compounds on mammary epithelial cells (MCF-10A) (Youssif, B. G. M. et al., 2019; Mahmoud, M. A., et al., 2022). The medium in which cells were propagated contained Dulbecco's modified Eagle's medium (DMEM)/ Ham's F-12 medium (1:1) supplemented with epidermal growth factor (20 ng/mL), hydrocortisone (500 ng/mL), insulin (10 μg/mL), 2 mM glutamine and 10% foetal calf serum. After every 2-3 days, the cells were passaged using trypsin ethylenediamine tetra acetic acid (EDTA). The cells were seeded at a density of $10^4$ cells $mL^{-1}$ in flat-bottomed culture plates containing 96 wells each. After 24 h, medium was removed from the plates and the compounds in (in 0.1% DMSO) were added (in 200 μL medium to yield a final concentration of 0.1% v/v) to the wells of plates. A single compound was designated with four wells followed by incubation of plates for 96h at 37°C. After incubation, medium was removed completely from the plates followed by addition of MTT (0.4 mg/mL in medium) to each well and subsequent incubation of plates for 3h. MTT (along with the medium) was removed and DMSO (150μL) was added to each well of the culture plates, followed by vortexing and subsequent measurement of absorbance (at 540 nm) using microplate reader. The data are shown as percentage inhibition of proliferation in comparison with controls containing 0.1% DMSO.

#### 4.2.1.2. Assay for antiproliferative effect

To explore the antiproliferative potential of compounds MTT assay was performed according to previously reported procedure (El-Sherief, H.A.M., et al., 2018; Abdelrahman, M. H., et al., 2017) using different cell lines to explore the antiproliferative potential of compounds propidium iodide fluorescence assay was performed using different cell lines. To calculate the total nuclear DNA, a fluorescent dye (propidium iodide, PI) is used which can attach to the DNA, thus offering a quick and precise technique. PI cannot pass through the cell membrane and its signal intensity can be considered as directly proportional to quantity of cellular DNA. Cells whose cell membranes are damaged or have changed permeability are counted as dead ones. The assay was performed by seeding the cells of different cell lines at a density of 3000-7500 cells/well (in 200µl medium) in culture plates followed by incubation for 24h at 37 °C in humidified 5% $CO_2$/95% air atmospheric conditions. The medium was removed; the compounds were added to the plates at 10 µM concentrations (in 0.1% DMSO) in triplicates, followed by incubation for 48 h. DMSO (0.1%) was used as control. After incubation, medium was removed followed by the addition of PI (25 µl, 50µg/mL in water/medium) to each well of the plates. At -80 °C, the plates were allowed to freeze for 24 h, followed by thawing at 25°C. A fluorometer (Polar-Star BMG Tech) was used to record the readings at excitation and emission wavelengths of 530 and 620 nm for each well. The percentage cytotoxicity of compounds was calculated using the following formula:

$$\%\ \text{Cytotoxicity} = \frac{A_C - A_{TC}}{A_C} \times 100$$

Where $A_{TC}$= Absorbance of treated cells and $A_C$= Absorbance of control. Erlotinib was used as positive control in the assay.

#### 4.2.1.3. EGFR inhibitory assay

EGFR-TK assay was performed to evaluate the inhibitory potency of the tested compounds against EGFR (Mohamed, F. A. M., et al., 2021). Baculoviral expression vectors including pBlueBacHis2B and pFASTBacHTc were used separately to clone 1.6 kb cDNA coding for EGFR cytoplasmic domain (EGFR-CD, amino acids 645–1186). 5′ upstream to the EGFR sequence comprised a sequence that encoded $(His)_6$. Sf-9 cells were infected for 72h for protein expression. The pellets of Sf-9 cells were solubilized in a buffer containing sodium vanadate (100 µM), aprotinin (10 µg/mL), triton (1%), HEPES buffer (50mM), ammonium molybdate (10 µM), benzamidine HCl (16 µg/mL), NaCl (10 mM), leupeptin (10 µg/mL) and pepstatin (10 µg/mL) at 0°C for 20 min at pH 7.4, followed by centrifugation for 20 min. To eliminate the non-specifically bound material, a Ni-NTA super flow packed column was used to pass through and wash the crude extract supernatant first with 10 mM and then with 100 mM imidazole. Histidine-linked proteins were first eluted with 250 and then with 500 mM imidazole after dialysis against NaCl (50 mM), HEPES (20 mM), glycerol (10%) and 1 µg/mL each of aprotinin, leupeptin and pepstatin for 120 min. The purification was performed either at 4 °C or on ice. To record autophosphorylation level, EGFR kinase assay was carried out based on DELFIA/Time-Resolved Fluorometry. The compounds were first dissolved in DMSO absolute, after dilution to appropriate concentration using HEPES (25 mM) at pH 7.4. Each compound (10 µL) was incubated with recombinant enzyme (10 µL, 5 ng for EGFR, 1:80 dilution in 100 mM HEPES) for 10 min at 25°C, after the addition of 5X buffer (10 µL, containing 2 mM $MnCl_2$, 100 µM $Na_3VO_4$, 20 mM HEPES and 1 mM DTT) and ATP-$MgCl_2$ (20 µL, containing 0.1 mM ATP and 50 mM $MgCl_2$) and incubation for 1h. The negative and positive controls were included in each plate by the incubation of enzyme either with or without ATP-$MgCl_2$. The liquid was removed after incubation and the plates were

washed thrice using wash buffer. Europium-tagged antiphosphotyrosine antibody (75 µL, 400 ng) was added to each well followed by incubation of 1h and then washing of the plates using buffer. The enhancement solution was added to each well and the signal was recorded at excitation and emission wavelengths of 340 at 615 nm. The autophosphorylation percentage inhibition by compounds was calculated using the following equation:

$$100\% - [(negative\ control)/(positive\ control) - (negative\ control)]$$

Using the curves of percentage inhibition of eight concentrations of each compound, $IC_{50}$ was calculated. Majority of signals detected by antiphosphotyrosine antibody were from EGFR because the enzyme preparation contained low impurities.

**4.2.1.4. BRAF kinase assay**

$V^{600E}$ mutant BRAF kinase assay was performed to investigate the activity of tested compounds against BRAF (Mohassab, A. M., et al., 2021). Mouse full-length GST-tagged $BRAF^{V600E}$ (7.5 ng, Invitrogen, PV3849) was pre-incubated with drug (1 µL) and assay dilution buffer (4 µL) for 60 min at 25°C. In assay dilution buffer, a solution (5 µL) containing $MgCl_2$ (30 mM), ATP (200 µM), recombinant human full length (200 ng) and *N*-terminal His-tagged MEK1 (Invitrogen) was added to start the assay, subsequent to incubation for 25 min at 25°C. The assay was stopped using 5X protein denaturing buffer (LDS) solution (5 µL). To further denature the protein, heat (70° C) was applied for 5 min. 4-12% precast NuPage gel plates (Invitrogen) were used to carry out electrophoresis (at 200 V). 10 µL of each reaction was loaded into the precast plates and electrophoresis was allowed to proceed. After completion of electrophoresis, the front part of the precast gel plate (holding hot ATP) was cut and afterwards cast-off. The dried gel was developed using a phosphor screen. A reaction without active enzyme was used as negative control while that containing no inhibitor served as positive control. To study the effect of compounds on cell-based

pERK1/2 activity in cancer cells, commercially available ELISA kits (Invitrogen) were used according to manufacturer's instructions.

### 4.3. Statistical analysis

Computerized Prism 5 program was used to statistically analyzed data using one-way ANOVA test followed by Tukey's as post ANOVA for multiple comparison at $P \leq .05$. Data were presented as mean ± SEM.

**Protocol of docking studies**

The automated docking simulation study is performed using Molecular Operating Environment (MOE®) version 2014.09. The X-ray crystallographic structure of the target EGFR and BRAF obtained from the protein data bank (PDB: 1M17, 5JRQ), respectively was obtained from Protein data bank. The target compounds were constructed into a 3D model using the builder interface of the MOE® program. After checking their structures and the formal charges on atoms by 2D depiction, the following steps were carried out: The target compounds were subjected to a conformational search. All conformers were subjected to energy minimization; all the minimizations were performed with MOE until a RMSD gradient of 0.01 Kcal/ mole and RMS distance of 0.1 Å with MMFF94X force-field and the partial charges were automatically calculated. The protein was prepared for docking studies by adding hydrogen atoms to the system with their standard geometry. The atoms connection and type were checked for any errors with automatic correction. Selection of the receptor and its atoms potential were fixed. MOE Alpha Site Finder was used for the active site search in the enzyme structure using all default items. Dummy atoms were created from the obtained alpha spheres.
